# Small-Molecule Host-Defense Peptide Mimetic Antibacterial and Antifungal Agents Activate Human and Mouse Mast Cells via Mas-Related GPCRs

**DOI:** 10.3390/cells8040311

**Published:** 2019-04-03

**Authors:** Ibrahim Alkanfari, Katie B. Freeman, Saptarshi Roy, Tahsin Jahan, Richard W. Scott, Hydar Ali

**Affiliations:** 1 Department of Pathology, University of Pennsylvania School of Dental Medicine, Philadelphia, PA 19104, USA; aibra@upenn.edu (I.A.); roysapta@upenn.edu (S.R.); Jtahsin@pennmedicine.upenn.edu (T.J.); 2 Fox Chase Chemical Diversity Center, Doylestown, PA 18902, USA; kfreeman@fc-cdci.com (K.B.F.); rscott@fc-cdci.com (R.W.S.)

**Keywords:** host-defense peptide mimetics, mast cells, MRGPRX2, Mrgprb2

## Abstract

Host-defense peptides (HDPs) have an important therapeutic potential against microbial infections but their metabolic instability and cellular cytotoxicity have limited their utility. To overcome these limitations, we utilized five small-molecule, nonpeptide HDP mimetics (smHDPMs) and tested their effects on cytotoxicity, antimicrobial activity, and mast cell (MC) degranulation. None of the smHDPMs displayed cytotoxicity against mouse 3T3 fibroblasts or human transformed liver HepG2 cells. However, one compound had both antifungal and antibacterial activity. Surprisingly, all five compounds induced degranulation in a human MC line, LAD2, and this response was substantially reduced in Mas-related G protein-coupled receptor (GPCR)-X2 (MRGPRX2)-silenced cells. Furthermore, all five compounds induced degranulation in RBL-2H3 cells expressing MRGPRX2 but this response was abolished in cells expressing naturally occurring loss-of-function missense variants G165E (rs141744602) and D184H (rs372988289). Mrgprb2 is the likely mouse ortholog of human MRGPRX2, which is expressed in connective tissue MCs (CTMCs) such as cutaneous and peritoneal MCs (PMCs). All five smHDPMs induced degranulation in wild-type PMCs but not in cells derived from Mrgprb2^−^/^−^ mice. These findings suggest that smHDPMs could serve as novel targets for the treatment of drug-resistant fungal and bacterial infections because of their ability to harness CTMCs’ host defense functions.

## 1. Introduction

Mast cells (MCs) are granulated immune cells of hematopoietic origin that are widely distributed in tissues such as the skin and mucosal tissues that interact with the environment. Although MCs are best known for their roles in IgE-mediated allergic reactions, their most important functions likely include tissue homeostasis, host defense, and wound healing [1,2,3,4]. MCs display considerable heterogeneity based on their tissue localization, protease composition of their secretory granules, development pattern, and cell surface receptor expression. In humans, skin MCs are known as MC_TC_ because their secretory granules contain both tryptase and chymase but those present in the lung are known as MC_T_ because their secretory granules contain only tryptase [5]. In rodents, skin MCs resemble human MC_TC_ and are referred to as connective tissue MCs (CTMCs) but lung MCs resemble human MC_T_ and are known as mucosal MCs (MMCs). Interestingly, CTCMs contain abundant heparin in their granules but MMCs do not.

MCs are sometimes referred to as constitutive (innate) and mucosal (adaptive) not only based on their tissue distribution and granularity, but also on their development patterns and expression of cell surface receptors [6,7]. Thus, innate MCs (MC_TC_ and CTMCs) are present constitutively in connective tissues, and are generally unaffected by T cells and express the cell surface receptor, Mas-related G protein-coupled receptor (GPCR) X2 (MRGPRX2 human) and Mrgprb2 (mouse) [8]. By contrast, adaptive MCs are found predominantly in intraepithelial tissues (lung, gut), are induced by T-cell-dependent inflammation, and do not express MRGPRX2/Mrgprb2 [8,9,10]. It has been proposed that innate/constitutive MCs contribute to tissue homeostasis and host defense to bacterial infection through their degranulation and the subsequent recruitment of neutrophils and dendritic cells [4,11,12,13,14]. 

Infections by fungal organisms are associated with a wide spectrum of diseases ranging from acute self-limiting manifestations in immunocompetent individuals to allergy and severe life-threatening infections in immunocompromised patients [15]. Recently, there has been a tremendous increase in the incidence of fungal infection, which is attributed to the overuse of prophylactic antifungal therapy and increased resistance to these drugs [16,17,18]. Antibiotics have been used for treatment of bacterial infections since the early 1900s but the emergence of multidrug-resistant strains of microbes poses a tremendous public health concern globally [19]. Thus, there is an urgent need to develop a novel therapy for the treatment of fungal infection and infection caused by antibiotic-resistant bacteria. 

Antimicrobial peptides (AMPs), also known as host-defense peptides (HDPs), represent an evolutionarily ancient mechanism of innate immunity found in both animal and plant kingdoms [20,21,22]. These peptides are cationic amphiphiles and provide protection against a variety of organisms including bacteria, fungi, and parasites [21,23]. The cationic charge on HDPs provides electrostatic attraction with the anionic surface of microbial membrane and the hydrophobic surface mediates entry into the membrane leading to their death. Selectivity of HDPs for microbial pathogens versus mammalian cells is attributed to differences in membrane charge, phospholipid composition, and the presence of unique cell membrane and cell wall protein components [24]. However, many HDPs that have antimicrobial activity in vitro have limited efficacy in vivo; this is likely due to the ability of negatively charged host proteins to neutralize the positive charges on these peptides [25]. 

Synthetic HDPs and those generated in transgenic plants harness MCs’ innate immune function by promoting their degranulation through MRGPRX2 [25,26]. HDPs also regulate angiogenesis and promote wound healing [27,28]. These novel mechanisms of action are attributed to the low risk of developing resistance and thus appear to provide ideal therapeutic targets. However, chemical synthesis of HDPs is prohibitively expensive and postsynthetic modifications such as cyclization, disulfide bond formation, and folding may be inadequate for optimal antimicrobial activity [29]. In addition, HDPs are metabolically unstable and display cytotoxicity and these properties have limited their clinical utility [30]. To overcome these limitations, a series of small-molecule HDP mimetics (smHDPMs) has been developed [30,31]. These compounds are relatively inexpensive to synthesize and have distinct advantages over HDPs in terms of stability, bioavailability, and low toxicity. Furthermore, these compounds exhibit potent activity against both bacteria and fungi [32]. However, the possibility that smHDPMs harness MCs’ immunomodulatory properties via inducing their degranulation has not been tested.

The purpose of this study was to determine if noncytotoxic synthetic smHDPMs that display antifungal and antibacterial activity promote human MC degranulation via MRGPRX2. Although Mrgprb2 was originally identified as the mouse ortholog of human MRGPRX2 in CTMCs [8], a recent study demonstrated that Mrgprb1, Mrgprb10, and Mrgprc11 are also expressed in MCs [33]. Another goal of this study was to determine which of the mouse Mas-related GPCRs contribute to degranulation in response to smHDPMs. Two naturally occurring missense MRPGRX2 variants, G165E and D184H, display loss-of-function phenotype for MC activation by a number of ligands including the HDP, human β-defensin-3 [34]. We also sought to determine the effects of MRGPRX2 missense mutation on MC activation by smHDPMs. The data presented herein suggest that smHDPMs that harness MCs’ immunomodulatory properties may serve as novel antifungal and antibacterial agents. However, individuals harboring MRGPRX2 missense mutations G165E and D184H may display susceptibility to infection because of their inability to harness MC-mediated immunity.

## 2. Materials and Methods

### 2.1. Mice

C57BL/6 (WT) mice were obtained from the Jackson Laboratory (Bar Harbor, ME). Mrgprb2^−/−^ mice in C57BL/6 background were generated by CRISPR-Cas9 mediated deletion of Mrgprb2 in CRISPR-Cas9 core facility of the University of Pennsylvania. Four pairs of guide RNA (GR1: CaccGCT GCTCCTATTCTGGTCAGG GGG and AaacCCTGACCAGAATAGGAGCAGC; GR2: Cacc GACACTGAGTGTCTATATGG AGG and AaacCCATATAGACACTCAGTGTC; GR3: Cacc GGTTGTAAAAATGGTCCACA CGG and AaacTGTGGACCATTTTTACA ACC; GR4: CaccGAATACTTTTTCTTATCCGTG TGG and AaacCACGGATAAGAA AAAGTATTC) specifically bind to exon2 of Mrgprb2 and were injected into the fertilized eggs to generate Mrgprb2^−^/^−^ mice. Genomic DNA was isolated from the WT and Mrgprb2^−^/^−^ mice using the Qiagen DNeasy Blood and Tissue Kit according to the manufacturer’s protocol and genotyped using the following primer pair: 

Forward ATGAGTGGAGATTTCCTAATCAAGAATCT

Reverse GCTCTGAACAGTTTCCAGTTCTTCAGGGT

The resulted PCR products were run in 1.5% agarose gel and visualized using Kodak 4000MM image station.

Mice were housed in pathogen-free cages on autoclaved hardwood bedding. Seven- to twelve-week-old male and female mice were used in this study. All experiments were approved by the Institutional Animal Care and Use Committee at The University of Pennsylvania.

### 2.2. Materials

All cell culture reagents and DNP-specific mouse IgE (SPE-7) were purchased from Invitrogen (Carlsbad, CA, USA). Recombinant murine interleukin-3 (IL-3), stem cell factor (SCF), and recombinant human SCF were purchased from Peprotech (Rocky Hill, NJ, USA). DNP-BSA and p-nitrophenyl-N-acetyl-β-D-glucosamine (PNAG) were from Sigma-Aldrich (St. Louis, MO, USA), Compound 48/80 was from AnaSpec (Fremont, CA, USA). Amaxa transfection kit (Kit V) was purchased from Lonza (Gaithersburg, MD, USA). PE anti-human MRGPRX2 antibody was purchased from Biolegend (San Diego, CA, USA). Polyclonal MRGPRX2 Ab was purchased from Novus Biologicals (Littleton, CO, USA). HRP-conjugated anti-rabbit IgG was from Cell Signaling Technologies (Danvers, MA, USA). West Pico Chemiluminescent Substrate was from Thermo Scientific (Rockford, IL, USA). DNeasy Blood and Tissue Kit was purchased from Qiagen (Germantown, MD, USA). QuikChange II Site-Directed Mutagenesis Kit was purchased from Agilent Genomics (Santa Clara, CA, USA). Plasmid encoding hemagglutinin (HA)-tagged human MRGPRX2 in pReceiver-MO6 vector was obtained from GeneCopoeia (Rockville, MD, USA). Antimicrobial peptidomimetics (compound 1, compound 2, compound 3, compound 4, and compound 5) were obtained from Fox Chase Chemical Diversity Center (Doylestown, PA, USA).

### 2.3. Fungus MIC Assay

Fungal strains included a clinical isolate of *Candida albicans* (ATCC GDH2346), *Aspergillus fumigatus* (ATCC MYA-3626), and *Aspergillus flavus* (ATCC 204304). Minimum inhibitory concentration (MIC) assays were carried out in 96-well plates using the Clinical and Laboratory Standards Institute (CLSI) method C27-A3 for *C. albicans* and M38-A2 for *Aspergillus* species [32]. smHDPMs, each in stock solutions of 10 mM in DMSO, were diluted in 50 µL RPMI/MOPS pH 7.0 in a 96-well plate and 50 µL of diluted yeast were added to each well. Final DMSO concentrations in the assay did not exceed 1%. The plate was then incubated at 35 °C for 48 h. The MIC was determined as the lowest concentration of an antimicrobial agent that substantially inhibits the growth of the organism. All MIC assays were performed in duplicate. 

### 2.4. Bacterial MIC Assay

smHDPMs were tested for antibacterial activities against three Gram-negative bacteria (*Escherichia coli* [ATCC 25922], *Pseudomonas aeruginosa* [ATCC 10145], and *Klebsiella pneumonia* [ATCC 13883]) and two Gram-positive bacteria (*Staphylococcus aureus* [ATCC 27660] and *Enterococcus faecalis* [ATCC 29212]) using the Hancock modified broth assay [35,36]. Three milliliters cation-adjusted Mueller–Hinton medium was inoculated with 20 µL of frozen bacterial stock and incubated at 37 °C on a shaker platform (250 rpm) overnight. The suspension was diluted to approximately 5 × 10^5^ cfu/mL and inoculated into a polypropylene (Costar) 96-well, round-bottom plate (90 µL volumes). Compound stock solutions were prepared in DMSO and serial twofold dilutions of compounds were made in 0.01% acetic acid, 0.2% bovine serum albumin directly in the wells of the polypropylene plate at 10 µL/well (final concentrations of 100, 50, 25, 12.5, 6.25, 3.13, 1.56, 0.78, 0.39, 0.19, 0.098, and 0.049 µg/mL). DMSO concentrations did not exceed 1% in the assay. All samples were done in duplicate. One set of control wells included broth-only samples with dilution buffer for testing sterility and providing blank values for the assay readings. Vehicle-control wells containing the bacterial suspension with DMSO (no compound) were also included. Following the overnight incubation (18 h), the cell growth was assessed by observing the presence of “acceptable growth”, defined by CLSI as a ≥2 mm button or definite turbidity. MIC was defined as the lowest concentration where acceptable growth is not observed.

### 2.5. Cytotoxicity Assays

Cytotoxicity (50% effective concentration, CC_50_) was determined against mouse 3T3 fibroblasts (ATCC CRL-1658) and human transformed liver HepG2 cells (ATCC HB-8065) using an MTS viability assay according to the manufacturer’s protocol (Promega CellTiter 96 aqueous nonradioactive cell proliferation assay). Briefly, 3T3 cells were seeded at 2 × 10^4^ cells/well in Dulbecco’s modified Eagle’s medium (DMEM) supplemented with 10% bovine calf serum and HepG2 cells were seeded at 3 × 10^4^ cells/well in MEM supplemented with 10% fetal bovine serum. After 24 h of growth, the culture medium was replaced with medium lacking serum, and eight two-folds dilutions of each of the five compounds were added. Compound stock solutions were prepared in methanol and final methanol concentrations in the assay did not exceed 10%. Following incubation for 1 h at 37 °C, compound solutions were removed and medium containing serum was replenished. Viability was determined by addition of the tetrazolium compound, MTS, and the electron coupling agent, PMS, and then incubation at 37 °C for 2 h (3T3 cells) or 3 h (HepG2 cells) followed by absorbance measurements at 490 nm [37]. The CC_50_ was calculated using GraphPad Prism software (nonlinear fit).

### 2.6. Mast Cell Culture

The human mast cell line, LAD2, was maintained in complete StemPro-34 medium supplemented with L-glutamine (2 mM), penicillin (100 IU/mL), streptomycin (100 μg/mL), and 100 ng/mL recombinant human stem cell factor (rhSCF). Hemidepletions were performed weekly with media containing rhSCF (100 ng/mL) [38]. Rat basophilic leukemia (RBL-2H3) cells were maintained as monolayer cultures in DMEM supplemented with 10% FBS, L-glutamine (2 mM), penicillin (100 IU/mL), and streptomycin (100 μg/mL) [39]. Peritoneal mast cells (PMCs) were obtained from 6–8 weeks old C57BL/6 and Mrgprb2^-/-^ (C57BL/6 background) mice using i.p. injection of 8 mL MC disassociation medium that was made of HBSS with 3% FCS and 10 mM HEPES. The cells were cultured in Iscove’s Modified Dulbecco’s Medium (IMDM) supplemented with 10% FCS, murine IL-3 (10 ng/mL), and murine SCF (30 ng/mL) [40]. After 48 h, the medium and the suspended cells were removed and replaced with fresh medium containing murine IL-3 (10 ng/mL) and murine SCF (30 ng/mL). The floating cells were then used for the experiment within 2–3 weeks [41]. Bone-marrow-derived mast cells (BMMCs) were harvested by flushing bone marrow cells from the femurs of C57BL/6 mice and culturing the cells for 4–8 weeks in IMDM supplemented with 10% FCS, murine IL-3 (10 ng/mL). The cells were then used within 4–8 weeks.

### 2.7. Lentivirus-Mediated Knockdown of MRGPRX2 in LAD2 Cells

Lentivirus generation was performed in HEK293T cells as per manufacturer’s instructions. Transduction of virus particles in LAD2 cells was performed as described previously [26]. Briefly, LAD2 cells (5 × 10^6^) in 3.5 mL of medium were mixed with 1.5 mL viral supernatant at 37 °C for 8 h. Cells were centrifuged and cultured in the virus-free medium. Antibiotic selection (puromycin 2 µg/mL) was initiated 16 h after post-treatment. Cells were used for the assay four days after the initiation of antibiotic selection. 

### 2.8. Western Blotting to Determine MRGPRX2 Expression

Cell lysates were prepared from scrambled control and MRGPRX2 shRNA transduced LAD2 cells in RIPA buffer and protein was quantified using BCA protein assay kit (Thermo Scientific). Protein was separated in SDS-PAGE (10 %), transferred in PVDF membrane, and incubated overnight with anti-MRGPRX2 antibody (1:500) in blocking buffer (5% skim milk in PBS). This was followed by incubation with HRP conjugated anti-rabbit IgG (1:1000) and development by West Pico Chemiluminescent Substrate. 

### 2.9. Transfection of RBL-2H3 Cells and Flow Cytometry

Cells (2 ×10^6^) were transfected with plasmids (2 µg/µL) encoding MRGPRX2 or MRGPRX2 missense mutants using the Amaxa kit V using Amaxa Nucleofector device according to the manufacturer’s protocol [34]. For stable transfection, cells were cultured in the presence of G-418 (1 mg/mL) and used within one month of transfection. For transient transfection, cells were used within 16–20 h after transfection. To detect MRGPRX2 expression, cells (1 × 10^6^) were incubated with the PE-conjugated anti-MRGPRX2 antibody, washed in FACS buffer, fixed, and analyzed on a BD LSR II flow cytometer [34]. 

### 2.10. Degranulation Assay

RBL-2H3 cells (5 × 10^4^), LAD2 cells (10 × 10^4^), and PMCs (5 × 10^3^), BMMCs (5 × 10^4^), were seeded into 96-well plates in a total volume of 50 μl HEPES buffer containing 0.1% bovine serum albumin (BSA) and exposed to ligands for 30 min. Cells without treatment were designated as control. In some experiments, cells were treated with Pertussis toxin (PTx). For total β-hexosaminidase release, unstimulated cells were lysed in 50 μl of 0.1% Triton X-100. Aliquots (20 μl) of supernatants or cell lysates were incubated with 20 μl of 1 mM p-nitrophenyl-N-acetyl-β- D-glucosamine for 1 h at 37 °C. The reaction was stopped by adding 250 μl of a 0.1 M Na_2_CO_3_/0.1 M NaHCO_3_ buffer and absorbance was measured at 405 nm.

### 2.11. Statistical Analysis

Data shown are mean ± SEM values derived from at least three independent experiments. Statistical significance was determined by nonparametric t-Test and one- or two-way ANOVA. Error bars represent mean ± SEM. Differences were considered statistically significant at a value * *p* ≥ 0.05, ** *p* ≥ 0.01, and *** *p* ≥ 0.001 and **** *p* ≥ 0.001. Data were analyzed by GraphPad Prism version 6.07.

## 3. Results

### 3.1. Antifungal and Cytotoxic Activities of smHDPMs (Compounds 1, 2, and 3) against Candida albicans, Aspergillus fumigatus, and Aspergillus flavus

**Figure 1:**
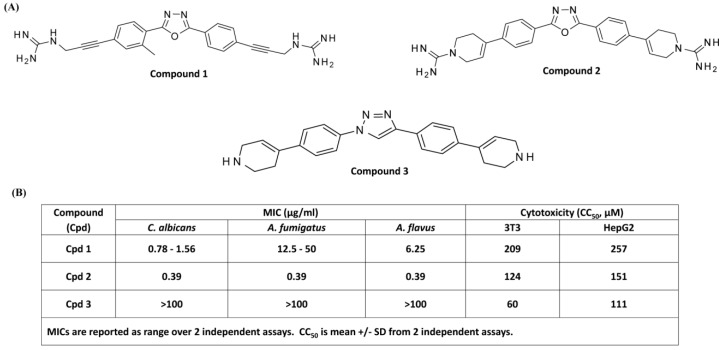
Structure and antifungal activity of three smHDPMs compounds (Cpd). (**A**) Structure of Cpd 1, Cpd 2, and Cpd 3. (**B**) Minimum inhibitory concentration (MIC) values (μg/mL) of smHDPMs against *C. albicans, A. fumigatus,* and *A. flavus.* Cytotoxicity of smHDPMs on 3T3 and HepG2 cell lines. CC_50;_ concentration of drug that reduces 50% cell viability.

HDPs are a diverse group of agents that are isolated from organisms across the phylogenetic spectrum. Despite this diversity, a hallmark of these peptides is that they display facially amphiphilic (FA) architecture in which the cationic groups and hydrophobic groups segregate into the opposite sides of the molecular backbone. It is thought that positive charges on the HDPs and anionic surface of microbial membrane provide recognition and the subsequent hydrophobic interaction perturbs membrane structure and function leading to microbial death [16]. A series of novel FA synthetic compounds based on a meta-phenylene backbone has been synthesized and tested for antimicrobial activity. Many of these compounds display antifungal activity in vitro and in vivo with little cytotoxic activity for mammalian cells [33,34,35]. We initially used three FA synthetic smHDPMs, namely compound 1, compound 2, and compound 3, with similar hydrophobic backbones but different cationic residues (Figure 1A). 

Minimal inhibitory concentrations (MICs) against *Candida albicans, Aspergillus fumigatus,* and *Aspergillus flavus* were determined. Compound 2 showed the highest antifungal activity with MIC value of 0.39 µg/mL. Compound 1 exhibited moderate antifungal activity against *Aspergillus fumigatus* with MIC value of 12.5–50 µg/mL but potent activity against *Candida albicans* and *Aspergillus flavus* with MIC values between 0.78 µg/mL and 6.25 µg/mL. By contrast, Compound 3 displayed poor antifungal activity with MIC > 100 µg/mL (Figure 1B). In order to determine the cytotoxicity of smHDPMs, we used two cell lines HepG2 and 3T3 cells and measured CC_50_ values. Compound 3 displayed cytotoxicity at concentrations similar to those required for antifungal activity. However, at concentrations that are relevant for antifungal activity, Compounds 1 and 2 displayed little to no cytotoxic effects on 3T3 and HepG2 cells (Figure 1B).

### 3.2. smHDPMs (Compounds 1, 2, and 3) Activate Human MCs via MRGPRX2

**Figure 2:**
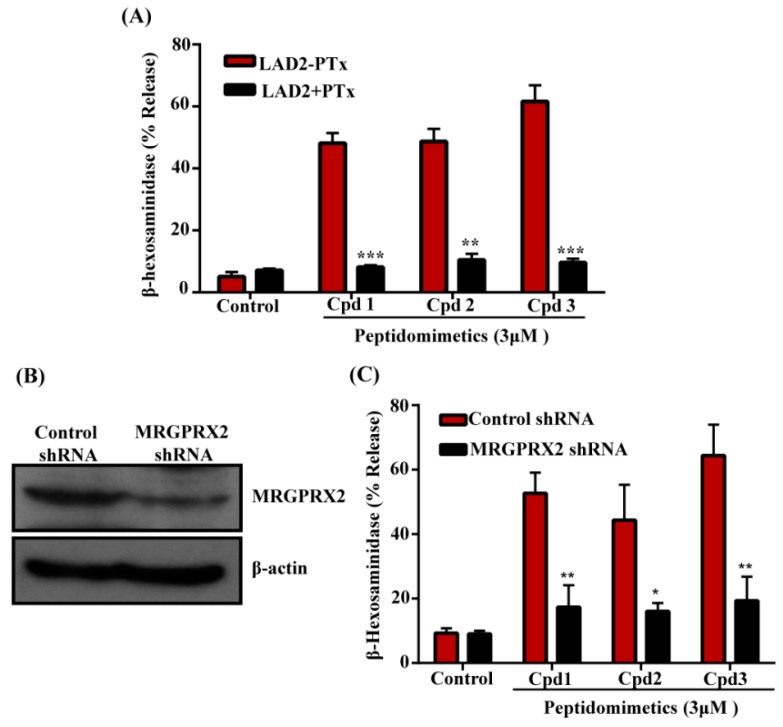
smHDPMs activate human mast cells via MRGPRX2. (**A**) LAD2 cells pretreated with or without pertussis toxin (PTx, 100 ng/mL, 16h) were exposed to vehicle (Control) or smHDPMs compounds (Cpd) 1, 2, and 3 (3 µM each) for 30 min and percentage of β-hexosaminidase release was measured. (**B**) Western blotting was performed to determine the expression level of MRGPRX2 in Control and MRGPRX2 knockdown LAD2 cells. (**C**) Control and knockdown cells were stimulated with smHDPMs Cpd 1, 2, and 3 (3 µM each) and percentage of β-hexosaminidase release was determined. All the points are expressed as a mean ± SEM of three experiments in triplicate. Statistical significance was determined by two-tailed unpaired t-Test. *** indicates *p* value <0.001,** indicates *p* value <0.01 and * indicates *p* value <0.05.

To determine if smHDPMs that display differences in antifungal activity stimulate human MCs, we tested the effects of Compounds 1, 2, and 3 on degranulation in a human MC line, LAD2, by quantitating the release of the enzyme β-hexosaminidase. Surprisingly, we found that while two of the three compounds had antifungal activity (Figure 1A), all three compounds induced robust β-hexosaminidase release (Figure 2A).

**Figure 3:**
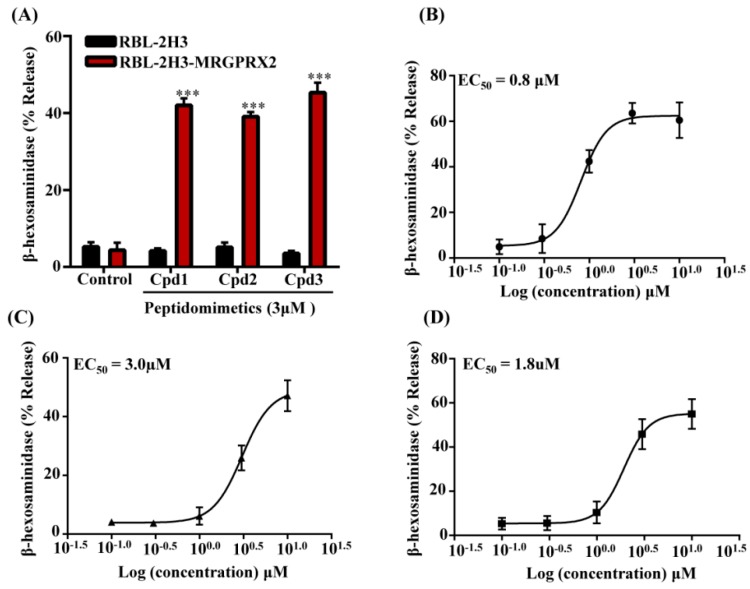
smHDPMs induce degranulation in RBL-2H3 cells expressing MRGPRX2. (**A**) Untransfected RBL-2H3 cells (RBL-2H3) and cells stably expressing MRGPRX2 (RBL-2H3-MRGPRX2) were exposed to vehicle (Control) or smHDPMs, (Cpd 1, 2, and 3, 3 µM) for 30 min and percentage of β-hexosaminidase release was determined. Concentration–response curves for (**B**) Cpd 1, (**C**) Cpd 2, and (**D**) Cpd 3 were determined using RBL-2H3-MRGPRX2 cells. Data are presented as a mean ± SEM and are representative of three independent experiments in triplicate. Statistical significance was determined by two-tailed unpaired t-Test. *** indicates *p* value <0.001.

To determine if the effects of these compounds are mediated via the activation of Gαi family of G proteins, we incubated cells with pertussis toxin (PTx). As shown in Figure 2A, degranulation in response to all three compounds was substantially inhibited by PTx, indicating the involvement of G proteins. HDPs induce degranulation in human MCs via MRGPRX2 [20,23,28]. To determine if these compounds also activate human MCs via MRGPRX2, we silenced its expression in LAD2 cells with lentiviral shRNA. Compared to the control-shRNA transduction, MRGPRX2-shRNA transduction resulted in a substantial reduction of MRGPRX2 expression as demonstrated by Western blotting (Figure 2B). Furthermore, degranulation induced by all three compounds was significantly inhibited in MRGPRX2-silenced cells when compared to control shRNA transduced cells (Figure 2C). RBL-2H3 is a rodent MC line that has been used to study IgE-mediated responses in vitro. Unlike LAD2 cells, it does not express MRGPRX2 and is unresponsive to its ligands. We therefore used RBL-2H3 cells stably expressing MRGPRX2 to confirm the role of this receptor on smHDPM-induced responses in MCs. As shown in Figure 3A, all compounds (3 µM) induced 40–50% degranulation in MRGPRX2-expressing cells but not in untransfected cells. RBL-2H3 cells expressing MRGPRX2 were used to determine the EC_50_ value for the smHDPMs. The EC_50_ values were 0.8 µM, 3 µM, and 1.8 µM for Compound 1, 2, and 3, respectively (Figure 3B–D).

### 3.3. Effects of Compounds 2, 4, and 5 on Antibacterial Activity and MC Degranulation

**Figure 4:**
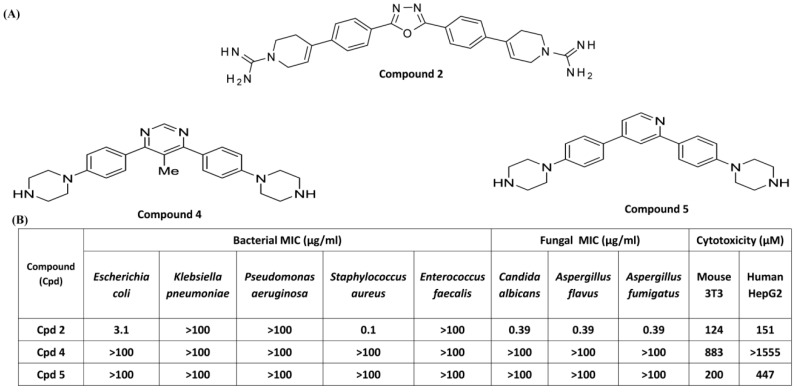
Structure and antibacterial activity of three smHDPMs compounds. (**A**) Structure of compound (Cpd) 2 and two of its derivatives, Cpd 4 and Cpd 5. (**B**) Minimum inhibitory concentration (MIC) values (μg/mL) of smHDPMs against three Gram-negative bacteria (*Escherichia coli, Pseudomonas aeruginosa, Klebsiella pneumonia*) and two Gram-positive bacteria (*Staphylococcus aureus and Enterococcus faecalis*). Cytotoxicity of smHDPMs on 3T3 and HepG2 cell lines. CC_50_ concentration of drug that reduces 50% cell growth.

We subsequently selected the most potent antifungal compound and its two derivatives for antibacterial screening against an array of bacteria (Figure 4A). Compound 2 showed potent antibacterial activities against one Gram-negative bacteria, *Escherichia coli*, and Gram-positive bacteria, *Staphylococcus aureus*, with MIC values of 3.1 and 0.1 µg/mL, respectively, which can be correlated with its potent antifungal activity (Figure 4B). However, this compound showed poor antibacterial activity against *Klebsiella pneumoniae*, *Pseudomonas aeruginosa, and Enterococcus faecalis*. 

**Figure 5:**
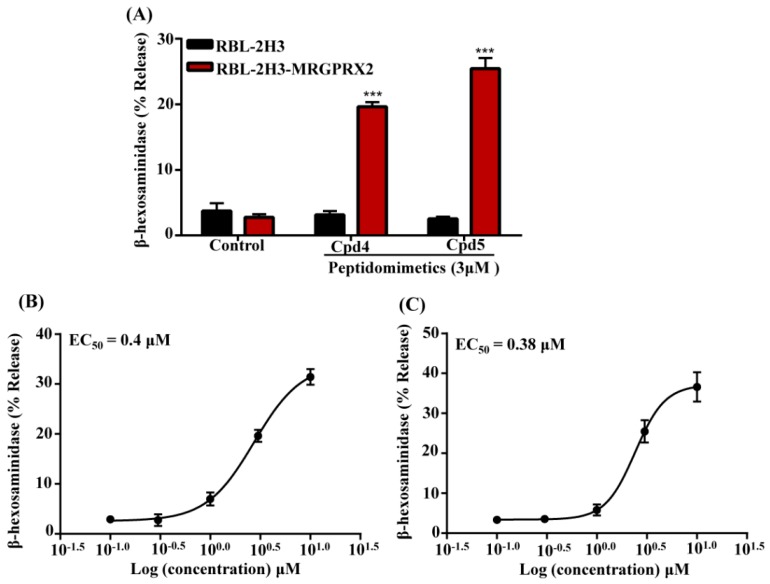
Compound 4 and Compound 5 induce degranulation in RBL-2H3 cells expressing MRGPRX2. (**A**) Untransfected RBL-2H3 cells (RBL-2H3) and cells stably expressing MRGPRX2 (RBL-2H3-MRGPRX2) were exposed to vehicle (Control), smHDPMs compound (Cpd) 4, and Cpd 5 (3 µM each) for 30 min and percentage of β-hexosaminidase release was determined. Concentration–response curves for (**B**) Cpd 4 and (**C**) Cpd 5 were performed using RBL-2H3-MRGPRX2 cells. Data are presented as mean ± SEM and are representative of three independent experiments in triplicate. Statistical significance was determined by two-tailed unpaired t-Test. *** indicates *p* value <0.001.

Two derivatives of Compound 2 (Compound 4 and Compound 5) showed poor antibacterial activity against all the bacterial strains tested (Figure 4B). Cytotoxicity of these three compounds was tested against two cells lines HepG2 and 3T3 cells. CC_50_ value of Compound 2 was 124 µM and 151 µM in mouse 3T3 and human HepG2 cells, respectively (Figure 4B). Compound 4 and 5 were 2–10-fold less cytotoxic than Compound 2. These compounds were then tested for their ability to activate MCs by MRGPRX2. We used RBL-2H3 cells stably expressing MRGPRX2 for these studies. As shown in Figure 5A, Compound 4 and Compound 5 (3 µM) induced 20–25 % degranulation in MRGPRX2-expressing cells but not in untransfected cells. Cells expressing MRGPRX2 were further used to determine the EC_50_ value for these smHDPMs. The EC_50_ values for Compounds 4 and 5 were 0.4 µM and 0.38 µM, respectively (Figure 5B,C). 

### 3.4. smHDPMs Activate Murine MCs via Mrgprb2

**Figure 6:**
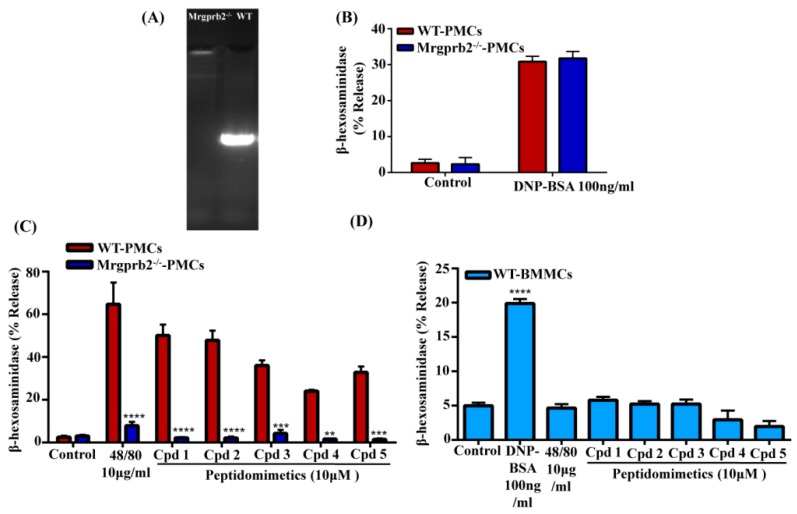
smHDPMs induce degranulation in PMCs via Mrgprb2. (**A**) Genotyping of wild-type (WT) and Mrgprb2^−^/^−^ by PCR using Mrgprb2-specific primers. (**B**) PMCs isolated from WT and Mrgprb2^−^/^−^ mice were exposed to IgE (1 µg/mL, 16 h) and then stimulated with antigen (DNP-BSA, 100 ng/mL, 30 min) and β-hexosaminidase release was determined. (**C**) PMCs were stimulated with compound 48/80 (10 µg/mL) or smHDPMs (Cps 1–5, 10 µM) for 30 min and β-hexosaminidase release was determined. (**D**) BMMCs were exposed to IgE (1 µg/mL, 16 h) and stimulated with antigen (DNP-BSA, 100 ng/mL), 48/80 (10 µg/mL), smHDPMs (10 µM) for 30 min and β-hexosaminidase release was determined. All the points expressed as a mean ± SEM of three experiments in triplicate. Statistical significance was determined by two-way and one-way ANOVA. **** indicates *p* value <0.0001, *** indicates *p* value <0.001 and ** indicates *p* value <0.01.

Although Mrgprb2 was originally identified as the mouse ortholog of human MRGPRX2 in CTMCs [8], a recent study demonstrated that Mrgprb1, Mrgprb10, and Mrgprc11 are also expressed in these MCs [33]. McNeil et al. [8] utilized zinc finger nuclease-based strategy to generate a mouse line with four-base-pair deletion in Mrgprb2 coding region (Mrgprb2^MUT^). For our studies, we used CRISPR/Cas9 technology to delete *Mrgprb2* in C57BL/6 mice. Deletion of the *Mrgprb2* was confirmed by genotyping (Figure 6A). Furthermore, as expected, the absence of Mrgprb2 in mouse PMCs had no effect on antigen/IgE-mediated degranulation (Figure 6B) but almost completely abolished the response to compound 48/80, a polymer known to activate MCs via Mrgprb2 (Figure 6C). All five smHDPMs induced degranulation in wild-type PMCs but this response was abolished in PMCs derived from Mrgprb2^−^/^−^ mice. BMMCs, which do not express Mrgprb2 [8], did not respond to compound 48/80 or any of the smHDPMs tested despite their normal responsiveness to antigen/IgE for degranulation (Figure 6D). These findings demonstrate that all five smHDPMs used in this study induce degranulation in mouse CTMCs via the activation of Mrgprb2. 

### 3.5. Naturally Occurring Missense MRGPRX2 Variants D184H, G165E Are Resistant to Activation by smHDPMs 

**Figure 7:**
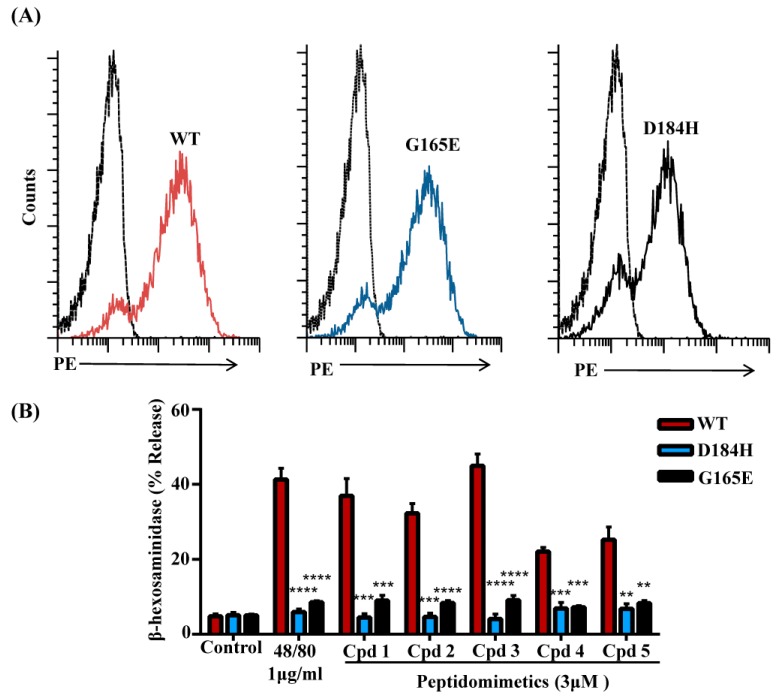
Missense MRGPRX2 variants do not respond to smHDPMs for degranulation. (**A**) RBL-2H3 cells were transiently transfected with cDNA encoding MRGPRX2 or its missense variants D184H and G165E. Receptor expression was determined by flow cytometry using anti-MRGPRX2-PE antibody. (**B**) RBL-2H3 expressing MRGPRX2 variants (D184H and G165E) were stimulated with smHDPMs (Cpds 1–5, 3.0 µM) for 30 min and β-hexosaminidase release was determined. All the points are expressed as a mean ± SEM of three experiments in triplicate. Statistical significance was determined by one-way ANOVA. **** indicates *p* value <0.0001,*** indicates *p* value <0.001 and ** indicates *p* value <0.01.

G protein-coupled receptors contain 7-transmembrane bundles that are connected by three extracellular loops (ECL1, ECL2, and ECL3) and three intracellular loops (ICL1, ICL2, and ICL3). The extracellular part also includes the N-terminus (N-term) and the intracellular (IC) part includes the helix VIII and a C-terminal sequence. GPCRs can be divided into modules; the EC and their closest TM regions have the greatest structural diversity and are responsible for the binding of diverse ligands. By contrast, the IC and its closest TM regions are responsible for G protein coupling and downstream signaling [36]. Recently, we screened eight naturally occurring missense variants within MRGPRX2’s ECL and TM domains from publicly available databases and found that two variants, D184H and G165E, displayed loss-of-function phenotype for activation by a number of ligands including the HDP, human β-defensin-3 [34]. We therefore sought to determine if these variants display loss-of-function phenotype for MC activation by smHDPMs. For this, RBL-2H3 cells were transfected with cDNAs encoding wild-type, D184H, and G165E variants. Flow cytometry analysis demonstrated that wild-type and mutant receptors are expressed on the cell surface at equivalent levels (Figure 7A). However, all five smHDPMs induced degranulation in cells expressing wild-type, but not the D184H or G165E variants (Figure 7B).

## 4. Discussion

The recent increase in the incidence of fungal infection has been attributed to the overuse of prophylactic antifungal therapy and increased resistance to these drugs [16,17,18]. Furthermore, although antibiotics have been used for the treatment of bacterial infections since the early 1900s, the emergence of multidrug-resistant strains of microbes poses a tremendous public health concern globally [19]. Thus, there is an urgent need to develop novel therapy for the treatment of infections caused by drug-resistant microbes. HDPs have important therapeutic potential against bacterial, viral, and fungal infections but their metabolic instability, poor tissue distribution, and cellular cytotoxicity have limited their utility [29,30]. To overcome these limitations, synthetic smHDPMs have been developed, which display broad-spectrum antimicrobial activity both in vitro and in vivo with low cytotoxicity [37,42,43,44,45]. Based on these findings, it has been proposed that smHDPMs could be developed as a new class of antifungal agents and antibiotics. The data presented herein raise the interesting possibility that, in addition to their direct antimicrobial activity, the therapeutic potential of smHDPMs reflects their ability to harness the host immune system via the activation of MCs through Mas-related GPCRs. 

Fungal skin infections are widespread and very common in humans [46]. Although MCs have been strongly implicated in antifungal host defense, their mechanisms remain largely unknown [46,47]. Fungal keratitis (FK), also known as keratomycosis or mycotic keratitis, is an infection caused by opportunistic *Fusarium*, *Aspergillus*, and *Candida albicans,* which are difficult to treat and may eventually require surgery [48,49]. MCs found in the corneal limbus are of the connective tissue (innate) type and they undergo degranulation in a mouse model of FK, resulting in vasodilation, increased intercellular adhesion molecule-1 (ICAM-1) expression on endothelial cells, and neutrophil infiltration [50]. Interestingly, stabilization of MCs with cromolyn leads to inhibition of MC degranulation, dramatic suppression of vascular dilation/permeability, lower ICAM-1 expression, and markedly reduced neutrophil infiltration, resulting in increased fungal growth and higher corneal perforation [50]. These findings provide strong support for the role of CTMCs (innate) in protecting the cornea against fungal infection through their degranulation and subsequent neutrophil recruitment. 

The mechanism via which corneal MCs undergo degranulation in FK is unknown but a role of neuropeptides has been proposed [50]. MRGPRX2 and Mrgprb2 are expressed predominantly in human and murine MCs, respectively, and are not found in any other immune or structural cells [8,10,51,52]. Given that neuropeptides such as substance P induce degranulation in MCs via MRGPRX2/Mrgprb2, this raised the interesting possibility that this receptor contributes to the role of MCs in host defense to fungal corneal infection [8,10,53]. It is noteworthy that in addition to histamine and proteases, MC granules release HDPs during degranulation, which further induces MC degranulation via MRGPRX2 [26,51,54,55]. These findings are consistent with the notion that noncytotoxic smHDPMs that display both antifungal activity and harness MCs’ immunomodulatory function may serve as a new class of antifungal agents. 

Our initial screen utilized three structurally related smHDPMs. We found that Compounds 1 and 2 displayed antifungal activity against *C. albicans, A. fumigitus,* and *A. flavus*. Compound 2 was more potent than Compound 1, but Compound 3 was inactive. At concentrations that are relevant for antifungal activity, Compounds 1 and 2 displayed little to no cytotoxic effects on 3T3 and HepG2 cells. Despite the difference in antifungal activity, we were surprised to find that all three smHDPMs induced strong degranulation in a human MC line, LAD2. The first indication that they induced MC degranulation via a GPCR was the finding that an inhibitor of Gαi family of G proteins completely blocked degranulation in response to all three smHDPMs. Our subsequent studies with shRNA-mediated gene silencing in LAD2 cells and MRGPRX2 transfected RBL-2H3 cells clearly showed that these smHDPMs induce degranulation of human MCs via MRGPRX2. The mouse counterpart of human receptor is Mrgprb2, which is expressed in PMCs but not BMMCs [8]. Our finding that smHDPMs induce degranulation in PMCs but not BMMCs supports our contention that these agents activate CTMCs via Mrgprb2. This contention was confirmed by the demonstration that these compounds did not induce degranulation in PMCs obtained from Mrgprb2^−^/^−^ mice. These findings suggest that two of the three smHDPMs used in our initial screen display both direct antifungal activity and harness MCs’ immunomodulatory property by inducing their degranulation and could serve as novel antifungal agents. 

In addition to fungal infection, MCs contribute to host defense against bacterial infection likely via MRGPRX2 and Mrgprb2. Thus, mastoparan, a peptide toxin isolated from wasp venom which has direct antibacterial activity, also induces degranulation in human and murine MCs via MRGPRX2 and Mrgprb2, respectively [8]. Arifuzzaman et al. [4] recently showed that mastoparan induces degranulation in mouse CTMCs to promote neutrophil recruitment, which accelerates bacterial (*S. aureus*) clearance in infected skin. Interestingly, a mastoparan derivative that does not induce MC degranulation but retains its antimicrobial activity against *S. aureus* is ineffective in clearing skin infection. By contrast, a mastoparan derivative that is devoid of antimicrobial activity against *S. aureus* but retains its ability to induce MC degranulation effectively clears skin infection. Based on these findings, it has been proposed that the therapeutic effect of mastoparan against *S. aureus* skin infection is attributed to its ability to induce MC degranulation rather than its direct antimicrobial activity [4]. CD301b+ dermal dendritic cells (DCs) promote re-epithelialization of sterile wounds and their numbers are decreased in *S. aureus*-infected skin [4,56]. However, MCs promote re-epithelialization by restoring the skin CD301b+ DC population. In addition, mastoparan boosts adaptive immunity and controls reinfection through the generation of cytokines from MCs [4]. These findings suggest that activation of Mrgprb2 in CTMCs by mastoparan not only promotes bacterial clearance by recruiting neutrophils but also facilitates skin regeneration and controls reinfection likely through the production of cytokines and the recruitment of DCs [4]. 

An interesting finding of the present study was that Compound 2 not only had broad-spectrum antifungal activity, it also effectively killed bacteria such as *E. coli* and *S. aureus*. However, its two structural derivatives with reduced positive charges (Compounds 4 and 5) did not display antibacterial activity. Despite this difference, all three compounds induced degranulation in LAD2 cells via MRGPRX2 and murine PMCs via Mrgprb2. Thus, similar to the case with mastoparan [4], Compounds 4 and 5 may promote *S. aureus* clearance from infected skin, facilitate skin regeneration, and control reinfection. There are, however, a number of important differences between mastoparan and smHDPMs. Thus, while EC_50_ values for human and mouse MC degranulation by mastoparan are between 25 µM and 50 µM [4], these values for smHDPMs are between 0.4 µM and 3 µM. This difference could reflect different affinities of mastoparan and smHDPMs for MRGPRX2/Mrgprb2 or that mastoparan is degraded by proteolytic enzymes released from MC granules, therefore requiring higher concentrations for MC degranulation. These findings have important implications for their effects on bacterial clearance and skin regeneration in vivo. It is likely that smHDPMs will promote these responses at lower concentrations than mastoparan. Brilacidin, a smHDPM structurally similar to the compounds used in the present study, is being developed for the treatment of acute bacterial skin infection [30]. Brilacidin also displays efficacy in a rabbit model of methicillin-resistant *S. aureus* (MRSA)-induced keratitis [57]. Based on the data presented in this study, and the in vivo findings with mastoparan and its analogs [4], it is possible that the potential clinical utility of brilacidin reflects its ability to activate MCs via MRGPRX2 in addition to its direct antimicrobial activities. 

One possible complication of using smHDPMs for controlling fungal and bacterial infections is that these agents could induce systemic anaphylaxis. This possibility is unlikely for the following reasons. First, MRGPRX2 and Mrgprb2 are expressed on “innate immune” types of MCs such as those found in the skin but not on “adaptive” types of MCs that are found in the lung and other mucosal tissues [8,9]. Second, topical application of mastoparan on *S. aureus*-infected mouse skin promotes both bacterial clearance and skin regeneration without any noticeable side effects at the application site or systemically [4]. Third, two peptide antibiotics approved by the US Food and Drug Administration, polymyxin B and colistin, activate MCs but do not induce anaphylaxis in patients [4,58]. One important finding of the present study was that MCs expressing missense MRGPRX2 variants G165E (rs141744602) and D184H (rs372988289) were resistant to degranulation in response to all smHDPMs tested [34]. Thus, if the potential clinical utility of smHDPMs reflects their ability to activate MCs via MRGPRX2, individuals harboring these mutations may be resistant to this type of therapy. 

In conclusion, we found that five smHDPMs with low cytotoxicity induced degranulation in human MCs via MRGPRX2 and murine MCs via Mrgprb2. These compounds displayed differences in their ability to kill bacteria and fungi. It is possible that these novel synthetic peptide mimetic MRGPRX2/Mrgprb2 agonists could form the basis of developing novel therapeutic agents for the treatment of drug-resistant fungal and bacterial infection via the harnessing of MCs’ immunomodulatory properties.
